# Mevalonate Diphosphate Decarboxylase MVD/Erg19 Is Required for Ergosterol Biosynthesis, Growth, Sporulation and Stress Tolerance in *Aspergillus oryzae*

**DOI:** 10.3389/fmicb.2019.01074

**Published:** 2019-05-16

**Authors:** Yunlong Sun, Yali Niu, Hui Huang, Bin He, Long Ma, Yayi Tu, Van-Tuan Tran, Bin Zeng, Zhihong Hu

**Affiliations:** ^1^Jiangxi Key Laboratory of Bioprocess Engineering and Co-Innovation Center for In-vitro Diagnostic Reagents and Devices of Jiangxi Province, College of Life Sciences, Jiangxi Science and Technology Normal University, Nanchang, China; ^2^National Key Laboratory of Enzyme – Protein Technology, VNU University of Science, Hanoi, Vietnam; ^3^Faculty of Biology, VNU University of Science, Hanoi, Vietnam

**Keywords:** mevalonate diphosphate decarboxylase, *Aspergillus oryzae*, sporulation, stress tolerance, ergosterol biosynthesis

## Abstract

Mevalonate diphosphate decarboxylase (MVD; EC 4.1.1.33) is a key enzyme of the mevalonic acid (MVA) pathway. In fungi, the MVA pathway functions as upstream of ergosterol biosynthesis, and MVD is also known as Erg19. Previously, we have identified *Aoerg19* in *Aspergillus oryzae* using bioinformatic analysis. In this study, we showed that AoErg19 function is conserved using phylogenetic analysis and yeast complementation assay. Quantitative reverse transcription–PCR (qRT-PCR) indicated that *Aoerg19* expression changed in different growth stages and under different forms of abiotic stress. Subcellular localization analysis showed that AoErg19 was located in the vacuole. Overexpression of *Aoerg19* decreased the ergosterol content in *A. oryzae,* which may due to the feedback-mediated downregulation of *Aoerg8*. Consistent with the decrease in ergosterol content, both *Aoerg19* overexpression and RNAi strains of *A. oryzae* are sensitive to abiotic stressors, including ergosterol biosynthesis inhibitor, temperature, salt and ethanol. Thus, we have identified the function of AoErg19 in *A. oryzae*, which may assist in genetic modification of MVA and the ergosterol biosynthesis pathway.

## Introduction

Mevalonate diphosphate decarboxylase (MVD; EC 4.1.1.33) is a key enzyme of the mevalonic acid (MVA) pathway (Krepkiy and Miziorko, 2004). It catalyzes the decarboxylation of the six-carbon mevalonate 5-diphosphate (MVAPP) to the five-carbon isopentenyl diphosphate (IPP) (Krepkiy and Miziorko, 2004), the basic structure required for the biosynthesis of isoprenoids, an important cellular intermediate (Bergès et al., 1997). MVD plays important roles in different organisms, including microbes, plants, and animals (Abbassi et al., 2015). In fungi, IPP is the precursor required for ergosterol biosynthesis and MVD is a crucial enzyme required for cell viability because the mutant of the corresponding gene was lethal at 35°C (Bergès et al., 1997). In higher plants, IPP is the basic skeleton required for the biosynthesis of isoprenoids, which is the precursor for many plant-specific molecules such as growth regulators (e.g., gibberellins and abscisic acid), photosynthetic pigments, phytotoxins, phytoalexins, and other compounds required for plant defense, as well as aromatic terpenoids and natural rubbers (Chappell, 1995; Shi et al., 2012; Abbassi et al., 2016). In rat, the reduced activity of MVD has been associated with lower serum cholesterol levels and subsequently severe hypertension and cerebral hemorrhage (Michihara et al., 1998).

Studies have shown that the function of MVD is conserved among many organisms as the genes encoding MVDs in *Candida albicans*, human, *Arabidopsis*, and *Ganoderma lucidum* can complement the temperature-sensitive phenotype of the *Saccharomyces cerevisiae mvd* mutant (Cordier et al., 1999; Dassanayake et al., 2002; Voynova et al., 2008; Shi et al., 2012). The enzymatic properties of MVD from several organisms have been identified. The decarboxylation catalyzed by MVD requires one molecule of ATP and the participation of Mg^2+^ (Bergès et al., 1997). The yeast two-hybrid assay showed that in human, rat, yeast, and Arabidopsis, MVD forms homodimers *in vivo;* it also revealed that MVD heterodimers can be formed between *S. cerevisiae* and Arabidopsis proteins, which further supported the evolutionarily conserved function of MVD (Cordier et al., 1999). The subcellular localization of MVD in different organisms has also been investigated. In the past decade, MVD was reported to be localized in peroxisome in human and rat cells (Kovacs et al., 2002). However, Hogenboom et al. (2004) showed that both endogenous human MVD and overexpressed MVD were localized in cytosol. Consistent with this result (Michihara et al., 2008) also showed that MVD is predominantly located in the cytosol of both B16 and B16F10 cells in mouse. Unlike animals, MVD has been reported to be localized in peroxisomes in plants (Clastre et al., 2011; Simkin et al., 2011). However, studies on the subcellular localization of MVD in fungi are limited.

In fungi, the MVA pathway acts as the upstream of ergosterol biosynthesis. Therefore, the *MVD* gene was also known as *erg19*. Ergosterol is a specific cell membrane component and has been widely used as a marker to assess fungal biomass (Beni et al., 2014). It is not only an important cell membrane component that affects fluidity and permeability, which plays important roles in fungal growth, reproduction and stress tolerance (Kodedova and Sychrova, 2015), but also an important pharmaceutical raw material that can be used as a precursor of vitamin D2, cortisone, and progesterone (Görög, 2011). Studies on yeast and other fungi have revealed that the ergosterol biosynthesis pathway involves 18 reactions and 24 enzymes in *S. cerevisiae* (Hu et al., 2017). It has been reported that *erg19* is one of the key genes in the ergosterol biosynthesis pathway (Bergès et al., 1997). The function of *erg19* in *S. cerevisiae*, *C. albicans* and other fungi has been studied. In *erg19* mutants, ergosterol biosynthesis was blocked, and the cell membrane composition was altered, which rendered the mutant temperature-sensitive (Cordier et al., 1999).

Till date, *erg19* in filamentous fungi has been scarcely studied. *Aspergillus oryzae*, one of the most important filamentous fungi with industrial applications, is a FDA and WHO identified safe production species. In addition to its long history in traditional food fermentation, and condiment and brewing industries in China, *A. oryzae* is increasingly being used in modern biotechnology industries such as enzyme and recombinant protein production (Yamada et al., 2014). Previously, we identified only one paralog of *erg19* in *A. oryzae* (named *Aoerg19*), as most of the other genes in the ergosterol biosynthesis pathway have multiple paralogs (Hu et al., 2019). Therefore, *erg19* may act as the key gene in ergosterol biosynthesis in *A. oryzae*. In this study, we determined the function, expression pattern, and subcellular localization of *AoErg19* and observed that both overexpression and RNAi of *Aoerg19* decreased ergosterol content in *A. oryzae* and changed the response of *A. oryzae* to abiotic stress agents.

## Materials and Methods

### Strains and Growth Conditions

*Aspergillus oryzae* 3.042 (CICC 40092) was obtained from the China Center of Industry Culture Collection (Beijing, China) and used as the wild type strain. The *A. oryzae* 3.042 aaapyrG was constructed using *Agrobacterium tumefaciens*-mediated transformation in our laboratory (Sun et al., 2019). *A. oryzae* was cultured on (Czapek-Dox Agar) medium (2% sucrose, 0.2% NaNO_3_, 0.1% KH_2_PO_4_, 0.05% MgSO_4_, 0.05% KCl, 0.05% NaCl, 0.002% FeSO_4_, 1.6% agar, pH 5.5) at 30°C. Spores were harvested after 3 days of culture by scraping the agar surface with a sterile glass spreader under a laminar flow hood and suspending the spores in sterile water containing 0.05% Tween-80. The concentration of spores was determined using a hemocytometer. *Escherichia coli* DH5α (Grant et al., 1990) was used for plasmid construction and *A. tumefaciens* AGL1 (Lazo et al., 1991) was used for genetic transformation of *A. oryzae*. Both *E. coli* and *A. tumefaciens* were cultured in LB medium supplemented with suitable antibiotics at 37°C and 28°C, respectively.

### DNA and RNA Extraction

The medium was covered with cellophane for DNA and RNA extraction. The mycelia were harvested and immediately frozen in liquid nitrogen and pulverized. DNA extraction was performed according to the instructions of the fungal DNA midi kit (Beyotime Institute of Biotechnology, Nantong, China). Total RNA was extracted from 0.5 g mycelia using a fungal RNA kit (Omega Bio-tek, Norcross, GA, United States) according to the manufacturer’s instructions, with the addition of an RNase-free DNase I treatment (Omega). The quality and concentration of the DNA and RNA samples were determined using NanoDrop ND-1000 spectrophotometer (Thermo Scientific, Wilmington, DE, United States).

### Expression Analysis of MVD

Total RNA was isolated from different growth stages or different stress conditions. The cDNAs were synthesized from 1 μg of total RNA using the Prime Script^TM^ RT reagent kit (Perfect Real Time; Takara). All quantitative reverse transcription–PCR (qRT–PCR) measurements were performed using a CFX96 real-time PCR detection system (Bio-Rad, CA, United States) with SYBR Premix Ex Taq (Takara, Japan), according to the manufacturer’s instructions. The house-keeping gene encoding histone H4 was used as a normalization control (Maruyama et al., 2002). The relative expression was calculated using the formula 2^-ΔΔCt^. The primer sequences for qRT–PCR are provided in Supplementary Table S1.

### Functional Complementation of Aoerg19 in Yeast

The *erg19* (Y41208) mutant was purchased from EUROSCARF^1^ and the yeast strain BY4741 was the wild-type control. Plasmid pYES2*-Aoerg19* contains *Aoerg19* full-length coding sequence (CDS) under the control of the *GAL1* promoter. To construct pYES2*-Aoerg19*, the CDS of *Aoerg19* was amplified using PCR (the primer sequences are listed in Supplementary Table S2) and *Hind*III and *Eco*RI restriction sites were added to facilitate cloning of the fragments into the pYES2 vector (Invitrogen, Shanghai, China). The recombinant plasmid was sequenced. Then, the pYES2*-Aoerg19* construct was transformed into the yeast mutant. The control and transformants were grown on YPD (1% yeast extract, 2% Bacto-Peptone, 2% glucose), and YPG (1% yeast extract, 2% Bacto-Peptone, 2% galactose) to assess the phenotypes.

### Overexpression and RNAi of AoErg19

To construct the overexpression vector, the CDS of *Aoerg19* was fused to DsRed at the C- and N-terminal of AoErg19 using fusion PCR, and the primers are listed in Supplementary Table S2. The binary vector pEX2B (Nguyen et al., 2017), was linearized with *Xho*I and *Bam*HI, followed by cloning of the fusion DNA fragments into pEX2B using a one-step cloning kit (Vazyme Biotech Co., Ltd., China) to construct pEX2B*-AoErg19-DsRed* and pEX2B*-DsRed-AoErg19* vectors. For the RNAi of *Aoerg19*, the 500 bp sequence of 5′ *Aoerg19* mRNA was cloned to construct the hairpin structure, and the 79 bp intron of α-amylase was used as the loop of the hairpin structure by three times PCR using RNAi-forward R1, R2, and R3. The primer sequences for RNAi are listed in Supplementary Table S2. All the constructed vectors were transformed into *A. tumefaciens* AGL1. Then, the vectors were transformed into *A. oryzae* 3.042 aaapyrG according to the procedure published by our laboratory (Sun et al., 2019). Single spores of the transformants were cultured for further analysis.

### Subcellular Localization of AoErg19

The constructed pEX2B-Aoerg19-DsRed and pEX2B-DsRed-Aoerg19 plasmids were transformed into *A. oryzae* 3.042 aaapyrG to determine the subcellular localization of the fusion protein. The pEX2B vector was transformed as a control. The peroxisome targeted signal (PTS1), SRL amino acid, was fused to the C-terminal of GFP using PCR to visualize peroxisomes (Escano et al., 2009). Then, the GFP sequence of pEX1-*ptr*A (Nguyen et al., 2016) was replaced with GFP-PTS1 to construct pEX1-*ptr*A-GFP-PTS1 (Sun et al., 2019). The first 72 amino acids of AoCit1 protein was used as the mitochondria targeted signal (MTS) (Takaya et al., 2009). The MTS encoded sequence was fused at the N-terminal of GFP by PCR (primers: MTS-F and MTS-R) to visualize mitochondria and the corresponding construct was named pEX1-*ptr*A-MTS-GFP. *Aspergillus nidulans* vacuolar carboxypeptidase Y (CPY) and *A. oryzae* vacuolar protein AoVam3 were used as vacuole maker by fusion the GFP at the C-terminal and N-terminal, respectively (Ohneda et al., 2002; Shoji et al., 2006). The CPY encoding sequences was synthetized by Sangon Biotech (Shanghai, China) and the corresponding construct was named pEX1-*ptr*A-CPY-GFP. The *Aovam3* sequence was amplified from the cDNA of *A. oryzae* using AoVam3-F and AoVam3-R primers and the corresponding vector was named pEX1-ptrA-GFP-AoVam3. For co-localization, these different GFP localization vectors were transformed into *A. oryzae* 3.042 aaapyrG, respectively. Then, pEX2B-Aoerg19-DsRed or pEX2B-DsRed-Aoerg19 was transformed into the transformants. The fluorescence was observed using confocal microscopy (Olympus FV1000MPE2). A 488 nm and 554 argon ion laser line was used for excitation of GFP and DsRed, while 505–515 nm and 585–595 nm emission filters were used for simultaneously capturing GFP and DsRed fluorescence, respectively, using the Olympus FV1000MPE2 confocal microscope. Primer sequences used for plastid construction were provided in Supplementary Table S2. For mitochondrial staining, mycelia grown on the slides were transferred into a medium containing Mito-tracker green (Beyotime Institute of Biotechnology, Nantong, China) and incubated for 30 min at 30°C (Tanabe et al., 2011). The mycelia were then washed twice with distilled water and observed using fluorescence microscopy.

### Measurement of Ergosterol, Mevalonic-5-PP and Isopentenyl-PP Content

Ergosterol extraction and quantification was performed according to Fowler et al. (2011). Briefly, the 48 h-old *A. oryzae* mycelium was harvested and vacuum freeze-dried to a constant weight. Then, the mycelium was pulverized into powder for the measurement of ergosterol, mevalonic-5-PP and isopentenyl-PP contents. Three milliliters of alcoholic KOH (25 g KOH plus 35 ml ddH_2_O, with 100% ethanol added to a total volume of 100 ml) was added to the 50 mg dry *A. oryzae* powder and mixed by vortexing for 1 min. The suspensions were then transferred to glass tubes and incubated at 85°C in a water bath for 1 h, after which the tubes were allowed to cool to ambient temperature. Three milliliters of n-heptane (Sigma-Aldrich, St Louis, MO, United States) was added to the tube, followed by vigorous vortexing for 3 min to extract ergosterol. The upper layer (the n-heptane layer) was isolated and stored at -20°C for 24 h prior to high-performance liquid chromatographic (HPLC) analysis. HPLC was conducted on Waters Alliance e2695 HPLC (Milford, MA) using a UV detector set at 282 nm with a Zorbax SB-C18 column. Methanol/water (95:5, v/v) was used as the mobile phase, and the elution rate was 1.5 ml min^-1^. Ergosterol (Sigma-Aldrich) was used to obtain a calibration curve. The measurement of mevalonic-5-PP and isopentenyl-PP content was performed by using ELISA kits (Qingdao Kechuang, China) according to the manufacturer’s instructions.

### Phylogenetic Analysis of MVD

The protein sequences of different organisms were collected from NCBI^2^. The IDs of the sequences are as follows: *Zea mays*-MVD1 (GI: 670375213), *Zea mays*-MVD2 (GI: 1142711841), *Oryza sativa*-MVD1(GI: 1443059899), *Oryza sativa*-MDV2 (GI: 1002245003), *Danio rerio* (GI: 55925435), *Mus musculus* (GI: 256985114), *Homo sapiens* (GI: 568815582), *Arabidopsis thaliana* (GI: 240254678), *S. cerevisiae* (GI: 330443715), *A. niger* (GI: 966767527), *A. oryzae* (GI: 391869300), *A. flavus* (GI: 238491808), *E. coli* (GI: 1205338453), *Lactobacillus composti* (GI: 737476048), and *Staphylococcus aureus* (GI: 739736044). Phylogenetic analysis was performed using the MEGA 5 software (Tamura et al., 2011). Briefly, the full-length amino acid sequences of these homologous genes were aligned using Muscle and a maximum likelihood tree was constructed using the alignment sequence with bootstrap value of 1000. Protein sequences were aligned using the DNAMAN software.

## Results

### AoErg19 Is Evolutionarily Conserved in Different Organisms

**FIGURE 1:**
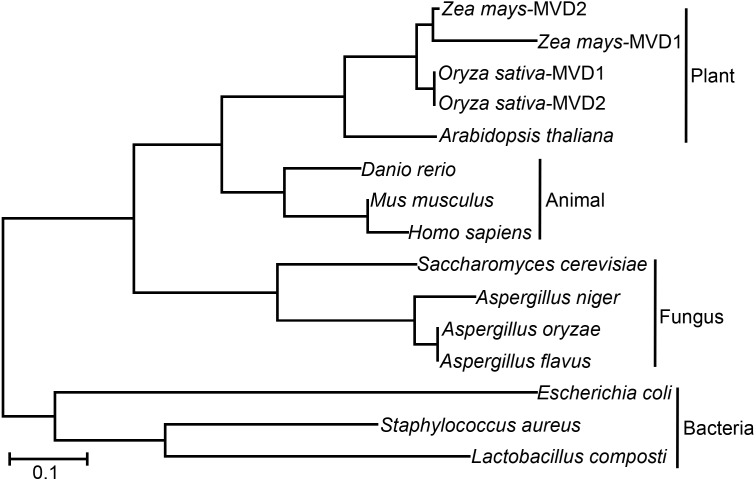
Phylogenetic analysis and amino acid sequence alignment of MVD in different species. Unrooted phylogenetic tree of MVD and homologous proteins in *Zea Mays*, *Oryza sativa*, *Danio rerio*, *Mus musculus*, *Homo sapiens*, *Arabidopsis thaliana*, *S. cerevisiae*, *A. niger*, *A. oryzae*, *A. flavus*, *E. coli*, *Lactobacillus composti,* and *Staphylococcus aureus*.

To investigate the function of MVD in *A. oryzae*, we performed phylogenetic analysis of MVD in different organisms. As shown in Figure 1, all the analyzed organisms contain at least one copy of *MVD*. The evolutionary relationships of the *MVD* genes are consistent with species evolution. For example, based on the origin of the *MVD* genes, the phylogenetic tree can be divided into plants, animals, fungi, and bacterial branches; in the fungi branch, the evolutionary distance of *Aspergillus* species (*A. niger*, *A. oryzae,* and *A. flavus*) is nearer than that of *S. cerevisiae*. However, MVD evolved two different paralogs in dicotyledons such as *Zea mays* and *Oryza sativa* (Figure 1). Protein alignment showed that MVD is conserved across plants, animals, fungi, and bacteria (Supplementary Figure S1), and the identity is 48%. *MVD* in the filamentous fungus *A. oryzae* is also known as *erg19* homolog in the yeast *S. cerevisiae*; therefore we named the homologous gene in *A. oryzae* as *Aoerg19*. These results indicate that *Aoerg19* is evolutionarily conserved across species.

### Aoerg19 Recovers the Phenotypes of the erg19 Mutant of *S. cerevisiae*

**FIGURE 2:**
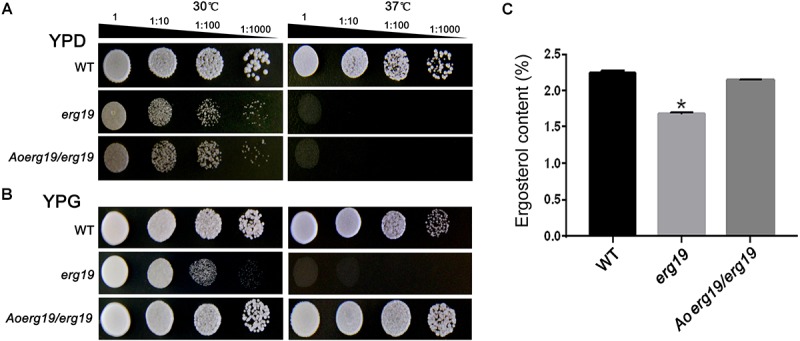
Phenotypes and ergosterol contents of *S. cerevisiae* with heterologous expression of *AoErg19*. **(A,B)** Growth of wild type *S. cerevisiae*, *erg19* mutant, and *AoErg19/erg19* transformant on YPD and YPG media. **(C)** Ergosterol content in wild type *S. cerevisiae*, *erg19* mutant, and *AoErg19/erg19* transformant. Asterisks denote significant differences compared to the control (*P* < 0.05).

Reports showed that the *S. cerevisiae* *erg19* (Y41208) mutant is temperature-sensitive (Dassanayake et al., 2002). To identify whether the function of *Aoerg19* is conserved, the full length CDS of *Aoerg19* was cloned into the yeast expression vector pYES2 and transformed into the *erg19* mutant. As the CDS of *Aoerg19* was under the *GAL1* promoter in the pYES2 vector, the transformants were grown on the YPD (with glucose) and YPG (with galactose as inducer) media at 30°C and 37°C to determine whether the temperature-sensitive phenotype of *erg19* was restored. Results showed that the *erg19* mutation was lethal at 37°C on YPD and YPG; *Aoerg19/erg19* can restore the lethal phenotype of the yeast on YPG (Figure 2A,B). Next, we detected the ergosterol content of these strains. Results showed that the *Aoerg19/erg19* transformants can recover the ergosterol deficiency of the *erg19* mutant (Figure 2C). Therefore, the function of AoErg19 is conserved between *S. cerevisiae* and *A. oryzae*.

### Aoerg19 Expression Differed at Different Growth Stages and Stress Environments

**FIGURE 3:**
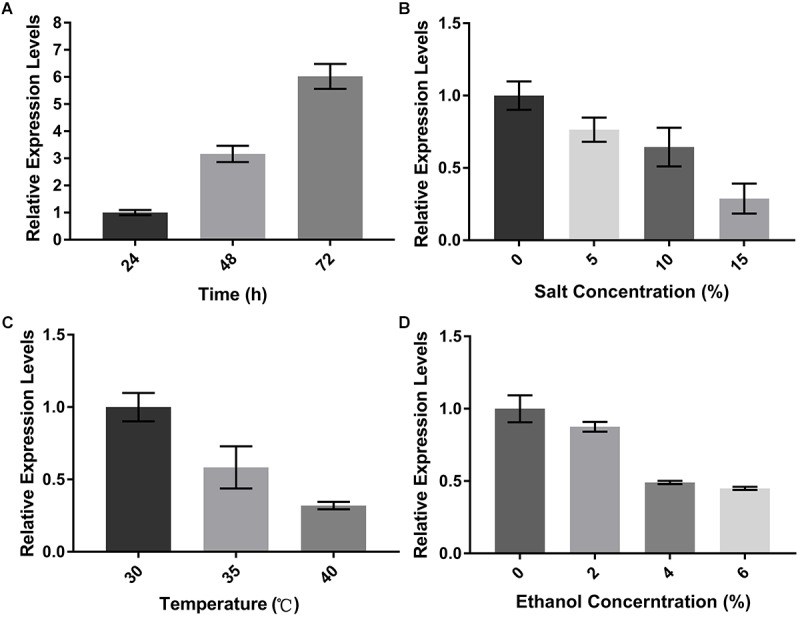
Expression levels of *AoErg19* at different developmental stages and under different abiotic stress. **(A)** Expression of *AoErg19* at 24, 48, and 72 h of growth; **(B–D)** Expression of *AoErg19* under salt, temperature, and ethanol stress conditions. The wild-type *A. oryzae* spore suspension was plated onto CD agar medium or CD agar medium supplied with NaCl or ethanol and incubated at 30°C (except temperature stress). For the determination of *AoErg19* mRNA levels in different growth stages, mycelia were harvested at 24, 48, and 72 h; for other tests, the mycelia were harvested at 48 h. Values represent the mean ± SD of three independent experiments.

To investigate the role of *Aoerg19* during *A. oryzae* growth, we determined the expression patterns of *Aoerg19* at different developmental stages using qRT-PCR. As shown in Figure 3A, the transcript level of *Aoerg19* increased during *A. oryzae*, and it was maximum in the 72 h-old mycelium. The colony morphologies of *A. oryzae* after 24, 48, and 72 h cultivation was shown as Supplementary Figure S2. Ergosterol is reported to be involved in stress responses in *S. cerevisiae* (Kodedova and Sychrova, 2015), therefore we also investigated the expression of *Aoerg19* under different stress conditions. *A. oryzae* was subjected to salt, temperature, and ethanol stress. Results showed that the *Aoerg19* transcript level decreased under these abiotic stress conditions; the transcript level of *Aoerg19* decreased with increase in salt and ethanol concentration and temperature (Figure 3B–D). Conclusively, the expression level of *Aoerg19* changed at different growth stages and under different forms of abiotic stress.

### AoErg19 Was Localized in the Vacuole

**FIGURE 4:**
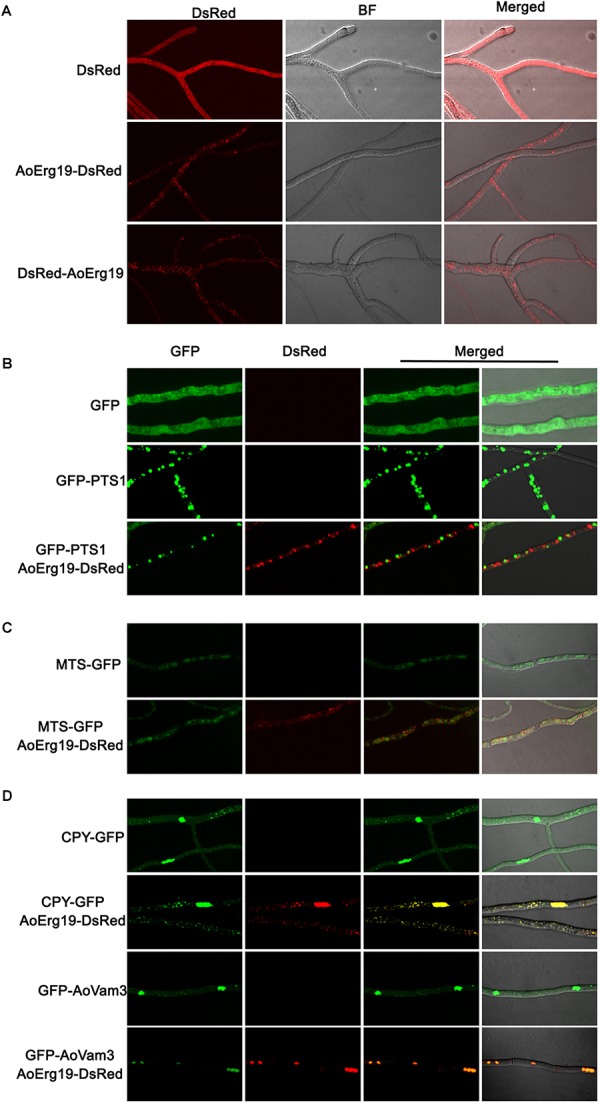
Subcellular localization of AoErg19. **(A)** The mycelium of *A. oryzae* 3.042 aaapyrG transformed with DsRed, AoErg19-DsRed, and DsRed-AoErg19 vectors. Left to right: fluorescent image of DsRed, bright field, and merged image of DsRed and bright field. **(B)** Co-localization of AoErg19-DsRed and peroxisome. The mycelium of *A. oryzae* 3.042 *ΔpyrG* transformed with GFP, GFP-PTS1, and co-transformed with GFP-PTS1 and AoErg19-DsRed. **(C)** Co-localization of AoErg19-DsRed and mitochondria. The mycelium of *A. oryzae* 3.042 *ΔpyrG* transformed with MTS-GFP and AoErg19-DsRed. **(D)** Co-localization of AoErg19-DsRed and vacuole. The mycelium of *A. oryzae* 3.042 *aaapyrG* transformed with CPY-GFP and GFP-AoVam3, and co-transformed with CPY-GFP or GFP-AoVam3 with AoErg19-DsRed, respectively. In **(B–D)**, left to right: fluorescent image of GFP, DsRed, merged image of GFP and DsRed, and merged image of GFP, DsRed and bright field (data not shown).

Mevalonate diphosphate decarboxylase has been reported to localize in cytosol or peroxisomes in plants or animals (Kovacs et al., 2002; Hogenboom et al., 2004; Michihara et al., 2008; Clastre et al., 2011; Simkin et al., 2011). However, studies on the subcellular localization of Erg19 homologs in filamentous fungi are limited. Bioinformatic analysis showed that there were no specific targeting signal sequences in the amino acid sequence of AoErg19 (data not shown). Therefore, we used DsRed as the reporter gene to investigate the subcellular localization of AoErg19 in *A. oryzae*. First, we fused the DsRed in the C-terminal and N-terminal region of AoErg19 to construct pYES2-*DsRed-Aoerg19* and pYES2-*Aoerg19-DsRed* vectors. Both vectors were transformed in the *S. cerevisiae* *erg19* mutant. Results showed that both DsRed-AoErg19 and AoErg19-DsRed can recover the temperature-sensitive phenotype and ergosterol deficiency of *erg19* mutant, indicating that the DsRed tag does not affect the function of AoErg19 (Supplementary Figure S3). Then, we transferred AoErg19-DsRed into pEX2B to construct *A. oryzae* transformation vectors. Both vectors were transformed into *A. oryzae* for the determination of AoErg19 subcellular localization. We observed that DsRed fluorescence displayed punctate structure with different sizes in the mycelium (Figure 4A). To further determine the organelle of AoErg19 localization, we investigated whether AoErg19 co-localized with peroxisomes, mitochondria or vacuole. The construct containing GFP-PTS1, MTS-GFP, CPY-GFP or GFP-AoVam3 was co-transformed with AoErg19-DsRed into *A. oryzae*, respectively. As shown in Figure 4B,C, the DsRed fluorescence was not coincident with GFP-PTS1 or MTS-GFP fluorescence, indicating that AoErg19 was not localized in peroxisomes or mitochondria. Mitochondria staining also confirmed that AoErg19 was not co-localized with mitochondria (Supplementary Figure S4). However, the DsRed fluorescence was coincident with both the two vacuole-localized GFP fluorescence (Figure 4D). We also performed the co-localization by co-transformation DsRed-AoErg19 with these different localized GFP vectors and find that AoErg19-DsRed was also co-localized with vacuole-localized GFP fluorescence (Supplementary Figure S5). Therefore, we concluded that AoErg19 was located in the vacuole.

### Overexpression and RNAi of Aoerg19 Affects the Sporulation and Ergosterol Content of *A. oryzae*

**FIGURE 5:**
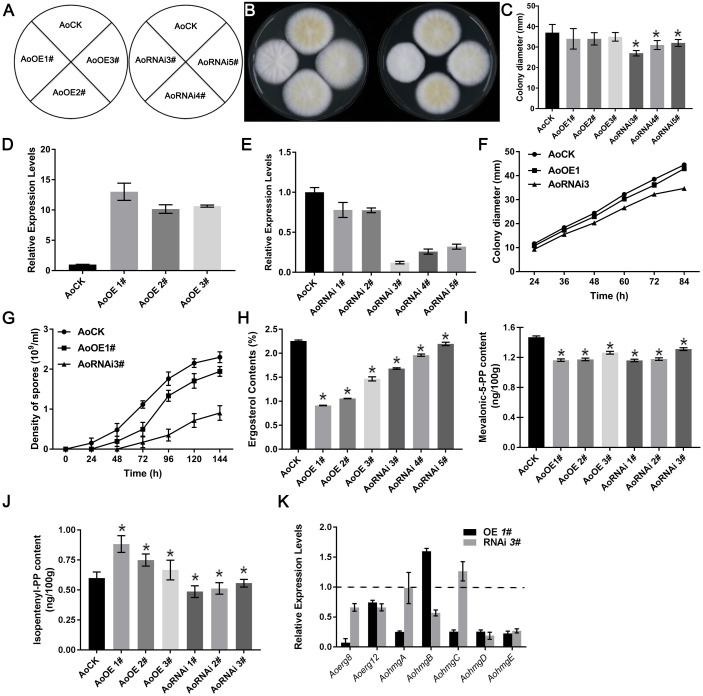
Ergosterol contents of *AoErg19* overexpression and RNAi strains. **(A)** Scheme showing different transgenic strains. **(B)** Colony morphologies of the control (AoCK, wild-type *A. oryzae*), overexpression and RNAi strains on the DPY media after 72 h of incubation. Spore suspension of identical concentrations of different *A. oryzae* strains were plated onto DPY agar medium and incubated at 30°C for 72 h. **(C)** Diameter of different transgenic strain colonies. **(D,E)** Relative mRNA levels of *AoErg19* in overexpression and RNAi strains. **(F)** The colony diameter of AoOE1# and AoRNAi3# at different growth time. **(G)** The spore number and sporulation time of AoOE1# and AoRNAi3#. **(H)** Ergosterol content in *Aoerg19* overexpression and RNAi strains. **(I)** Mevalonic-5-PP contents in *AoErg19* overexpression and RNAi strains. **(J)** Isopentenyl-PP contents in *AoErg19* overexpression and RNAi strains. **(K)** Expression levels of *Aoerg8*, *Aoerg12*, and *Aohmgs* in AoOE1# and AoRNAi3#. The colony diameter was calculated using the crisscross method, in which three independent experiments were performed. For qRT-PCR, the mycelia were harvested at 48 h; values represent the mean ± SD of three independent experiments. Asterisks denote significant differences compared to the control (*P* < 0.05).

We took advantage of the overexpression vector and the decrease of the target gene expression by RNAi to determine the function of *Aoerg19* in *A. oryzae*. As shown in Figure 5, the colony diameters of AoErg19 overexpression strains were not significantly different from that of the control; however, the RNAi strains were smaller (Figure 5A–C). The mRNA levels of *Aoerg19* in three overexpression strains and five RNAi strains were detected to confirm the effect of overexpression and RNAi. We observed that both overexpression and RNAi worked efficiently (Figure 5D,E). Then, the AoOE1# and AoRNAi3# were selected for further identification. The growth rate of AoOE1# colony was not significantly different from that of the control; however, the growth rate of AoRNAi3# was significantly lower (Figure 5F). Quantitation of the spore number and sporulation time of AoOE1# and AoRNAi3# showed that the spore number of both AoOE1# and AoRNAi3# decreased, whereas sporulation time of both increased (Figure 5G).

Because *Aoerg19* acts as the upstream of the ergosterol biosynthesis pathway, impairment or overexpression of *erg19* decreased ergosterol content in *S. cerevisiae* (Bergès et al., 1997). Hence, we detected the ergosterol contents of strains AoOE1#-AoOE3# and AoRNAi3#-AoRNAi5#. As expected, the ergosterol contents were reduced in the RNAi strains (Figure 5H). However, the ergosterol contents also decreased significantly in the overexpression strains, and were even lower than those of the RNAi strains (Figure 5H). We also detected the contents of the direct substrate (mevalonic-5-PP) and product (isopentenyl-PP) of AoErg19 in both OE and RNAi *A. oryzae* strains. The results showed that in both OE and RNAi strains, mevalonic-5-PP was decreased (Figure 5I); in OE strains, isopentenyl-PP contents increased while the isopentenyl-PP content was decreased in RNAi strains (Figure 5J). To investigate whether the decrease in ergosterol content in the overexpression strains was caused by feedback regulation, we used qRT-PCR to assess the expression of genes located in the upstream of *Aoerg19* (*Aoerg8*, *Aoerg12*, and *Aohmgs*) in the ergosterol biosynthesis pathway. Results showed that with the exception of *AohmgB*, the expression of these genes was reduced in AoOE1#, and *Aoerg8* showed the maximum reduction (Figure 5K), indicating that *AoErg8* is the feedback target in the *Aoerg19* overexpression strains; of the expression of these genes was lowered in AoRNAi3#, with the exception of *AohmgC* (Figure 5K). Taken together, these results showed that both up- and down-regulation of *eAoerg19* mRNA levels in *A. oryzae* impair sporulation and decrease ergosterol content.

### Aoerg19 Overexpression and RNAi Strains Are More Sensitive to Abiotic Stress

**FIGURE 6:**
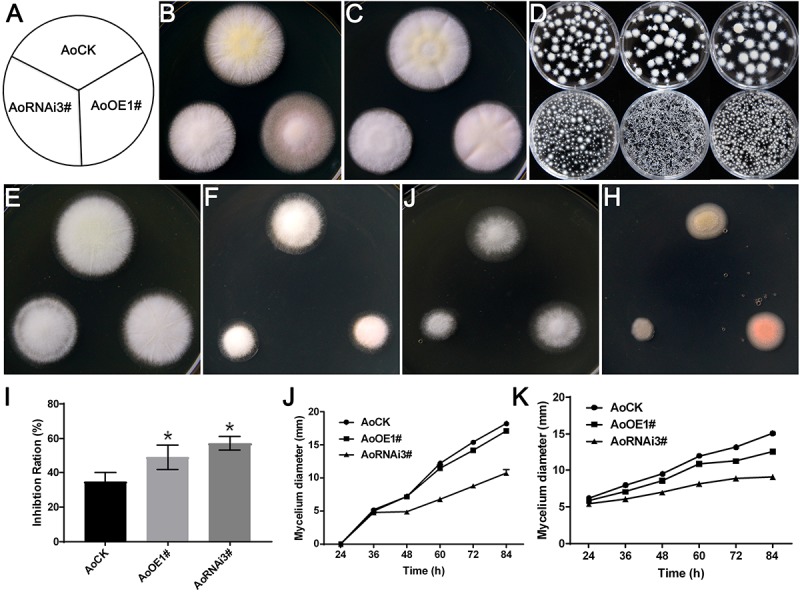
Colony morphologies of control, AoOE1#, and AoRNAi3# strains under different stress conditions. **(A)** Scheme showing the different *A. oryzae* strains in Figure 6. **(B,C)** The colony of control, overexpression, and RNAi strains cultured for 60 h on DPY media at 30 and 37°C. **(D)** Phenotypes of control, overexpression, and RNAi strains at 30°C (upper panel) and 37°C (lower panel) in liquid DPY media, from left to right: AoCK, AoOE1#, AoRNAi3#. **(E–H)** Phenotypes of control, AoOE1#, and RNAi3# strains cultured on DPY, DPY with 0.35 μg/L terbinafine, DPY with 10% NaCl, and DPY with 4% ethanol. **(I)** The inhibition ratio of terbinafine on the growth of control, AoOE1#, and RNAi3# stains. **(J,K)** The diameter of different strains cultured on 10% NaCl and 4% ethanol media at different time points. Asterisks denote significant differences compared to the control (*P* < 0.05).

Ergosterol deficiency in *S. cerevisiae* rendered it more sensitive to ergosterol biosynthesis inhibitor (Zuzana et al., 2013), and overexpression of *erg19* increased sensitivity to NaCl stress (Bhattacharya et al., 2018). Therefore, we investigated the growth of *Aoerg19* overexpression and RNAi strains to different abiotic stresses, including ergosterol biosynthesis inhibitor, temperature, salt, and ethanol. The differences among the three colonies on the DPY media were not obvious at 37°C (Figure 6B). However, we observed that under liquid culture conditions, the hyphal pellets of the three strains showed different phenotypes. For example, at 30°C, all the three strains can form hyphal pellets, although their shapes were slightly different (Figure 6D); the hyphal pellets were smaller at 37°C; however, the hyphal pellets of AoOE1# and AoRNAi3# strains were considerably smaller than that of the control (Figure 6D). The AoOE1# and AoRNAi3# strains were more sensitive than the control to the ergosterol biosynthesis inhibitor, temperature, salt, and ethanol treatment (Figure 6E–K and Supplementary Figure S6). Interestingly, the color of the overexpression colony turned red under ethanol stress (Figure 6H) which may be because of the accumulation AoErg19-DsRed protein. Thus, overexpression and RNAi of *Aoerg19* appear to increase sensitivity to different abiotic stress conditions.

## Discussion

The identification of genes involved in MVA pathway is important for genetic modification of MVA pathway, which has potential application for producing isoprenoids, an important feed-stock for commercial production of synthetic rubber and a high energy biofuel (Ma et al., 2011; Miziorko, 2011). Bioisoprene is synthesized by isoprene synthase from dimethylallyl diphosphate (DMAPP), the direct biosynthesis product of MVD (Hu et al., 2017). Meanwhile, in fungi, the MVA pathway is the upstream of ergosterol biosynthesis pathway, which has been well studied in *S. cerevisiae* (Hu et al., 2019). However, there was little research about ergosterol biosynthesis pathway in *A. oryzae.* Our previous study revealed that the ergosterol biosynthesis pathway is conserved between *S. cerevisiae* and *A. oryzae* by bioinformatics analysis. However, the ergosterol biosynthesis genes in *A. oryzae* are more complicated as there are multiple paralogs encoding the same biosynthetic enzymes (Hu et al., 2019). The research of *Aoerg19* does not only of significance in understanding MVA pathway but also in ergosterol biosynthesis pathway.

### The Function of AoErg19 in *A. oryzae*

The MVA pathway was conserved among different species (Jonathan and David, 2011). The function of MVD (AoErg19) in *A. oryzae* has not been identified. In this study, phylogenetic analysis and yeast complementation assay suggested that MVD function was conserved across fungal species (Figure 1, 2). The *Aoerg19* overexpression and RNAi strains of *A. oryzae* showed delayed sporulation and sensitive to abiotic stress, indicating that ergosterol is involved in sporulation and stress response. Besides, we have noticed that under ethanol stress, the color of the overexpression colony turns red, which may be caused by the accumulation the AoErg19-DsRed protein, indicating that ethanol stress can also affect the accumulation of AoErg19. The subcellular localization of MVD in different species has been studied and it was reported to vary in plants and animals. In plants MVD was located in peroxisomes, whereas in animals it was mainly located in the cytosol (Kovacs et al., 2002; Hogenboom et al., 2004; Michihara et al., 2008; Clastre et al., 2011; Simkin et al., 2011). However, the MVD of filamentous fungi have been scarcely studied. Here, we demonstrated that MVD in *A. oryzae* was located in the vacuole (Figure 4). The different subcellular localization of MVD among plants, animals, and fungi can provide some information for genetic modification of MVA pathway across different species.

### Ergosterol Content in Aoerg19 Overexpression and RNAi Strains

Ergosterol is a specific cell membrane component that is not only an important cell membrane component that affects fluidity and permeability, but also plays important roles in fungal growth, reproduction, and stress tolerance (Kodedova and Sychrova, 2015). Usually, ergosterol content is strictly regulated to appropriate levels by feedback regulation of ergosterol synthesizing activities at the transcriptional, translational, and posttranslational levels (Espenshade and Hughes, 2007). In yeast, many rate-limiting reactions in ergosterol biosynthesis have been identified. For example, the HMG is the first rate-limiting enzyme; overexpression HMG in *S. cerevisiae* resulted in squalene accumulation, but did not affect the content of ergosterol (Polakowski et al., 1998). Other enzymes such as Erg3, Erg4, Erg11, and Erg27 are also reported to be the possible rate-limiting enzyme in *S. cerevisiae* (Lees et al., 1995; Arthingtonskaggs et al., 1996; Veen et al., 2003). In filamentous fungi, there are also some researches about ergosterol biosynthesis, and main of them focused on fungicide discovery or drug resistance by using ergosterol biosynthesis inhibitors (Alcazar-Fuoli and Mellado, 2012; Shao et al., 2016; Dhingra and Cramer, 2017). Little of them focused on the function identification of these genes.

In *A. oryzae*, the research about MVA or ergosterol pathway is of great significance because *A. oryzae* is the GRAS microorganism and has broadly applied in industry. Our previous studies showed that ergosterol biosynthesis in *A. oryzae* is more complicated than in *S. cerevisiae* (Hu et al., 2019). In this study, we revealed that overexpression of *Aoerg19* resulting in the decreased ergosterol content by feedback regulation of *Aoerg8* (Figure 5H). The intermediate, mevalonic-5-PP was decreased in both OE and RNAi *A. oryzae* strains which was possible the comprehensive results of the gene expression of *Aoerg8* and *Aoerg19* (Figure 5I). Consistent with the function of ergosterol in fungal growth, reproduction, and stress tolerance, the decrease in ergosterol content in both overexpression and RNAi strains rendered *A. oryzae* more sensitive to abiotic stress (Figure 6). We observed that although the ergosterol content of the overexpression strain was lower than that of the RNAi strain, the RNAi stains are more sensitive to the abiotic stress (Figure 6). One explanation is that the ergosterol content in the overexpression strains was reduced by feedback regulation, and under stress conditions, the feedback regulation adjusted the ergosterol level to a suitable level for survival; alternatively, the accumulation of IPP, geranyl diphosphate, or FPP in the overexpression strains can act as anti-stress agents that assist in adapting to stress conditions.

## Conclusion

In summary, this study determined the function, expression pattern, and subcellular localization of *Aoerg19*. Overexpression of *Aoerg19* decreased ergosterol content, suggesting *Aoerg19* is the rate-limiting enzyme in the sterol biosynthesis pathway of *A. oryzae*. Further studies about the regulation network in ergosterol biosynthesis can be done to uncover the feedback regulation of MVA and ergosterol pathway. Thus, the gene function identification of *Aoerg19* in *A. oryzae* may provide some useful information for the genetic modification of MVA pathway in *A. oryzae*.

## Author Contributions

ZH and BZ designed the study and wrote the manuscript. YS and YN performed the main experiments. HH participated in the subcellular localization of AoErg19. BH and LM participated in the analysis of sterols. YT participated in the confocal microscopy. V-TT contributed to technical support of *A. oryzae* transformation and manuscript revision.

## Conflict of Interest Statement

The authors declare that the research was conducted in the absence of any commercial or financial relationships that could be construed as a potential conflict of interest.

## Footnotes

1 http://www.euroscarf.de/index.php

2 http://www.ncbi.nlm.nih.gov/

## References

[B1] Abbassi S. Patel K. Khan B. Bhosale S. Gaikwad S. (2016). Functional and conformational transitions of mevalonate diphosphate decarboxylase from *Bacopa monniera*. *Int. J. Biol. Macromol.* 83 160–170. 10.1016/j.ijbiomac.2015.11.067 26657583

[B2] Abbassi S. J. Vishwakarma R. K. Patel P. Kumari U. Khan B. M. (2015). *Bacopa monniera* recombinant mevalonate diphosphate decarboxylase: biochemical characterization. *Int. J. Biol. Macromol.* 79 661–668. 10.1016/j.ijbiomac.2015.05.041 26027607

[B3] Alcazar-Fuoli L. Mellado E. (2012). Ergosterol biosynthesis in aspergillus fumigatus: its relevance as an antifungal target and role in antifungal drug resistance. *Front. Microbiol.* 3:439. 10.3389/fmicb.2012.00439 23335918 PMC3541703

[B4] Arthingtonskaggs B. A. Crowell D. N. Yang H. Sturley S. L. Bard M. (1996). Positive and negative regulation of a sterol biosynthetic gene (ERG3) in the post-squalene portion of the yeast ergosterol pathway. *Febs Lett.* 392 161–165. 10.1016/0014-5793(96)00807-1 8772195

[B5] Beni A. Soki E. Lajtha K. Fekete I. (2014). An optimized HPLC method for soil fungal biomass determination and its application to a detritus manipulation study. *J. Microbiol. Meth.* 103 124–130. 10.1016/j.mimet.2014.05.022 24918988

[B6] Bergès T. Guyonnet D. Karst F. (1997). The *Saccharomyces cerevisiae* mevalonate diphosphate decarboxylase is essential for viability, and a single Leu-to-Pro mutation in a conserved sequence leads to thermosensitivity. *J. Bacteriol.* 179 4664–4670. 10.1128/jb.179.15.4664-4670.1997 9244250 PMC179309

[B7] Bhattacharya S. Esquivel B. D. White T. C. (2018). Overexpression or deletion of ergosterol biosynthesis genes alters doubling time, response to stress agents, and drug susceptibility in *Saccharomyces cerevisiae*. *mBio* 9:e1291–18. 10.1128/mBio.01291-18 30042199 PMC6058291

[B8] Chappell J. (1995). Biochemistry and molecular biology of the isoprenoid biosynthetic pathway in plants. *Annu. Rev. Plant Phys.* 46 521–547. 10.1146/annurev.pp.46.060195.002513

[B9] Clastre M. Papon N. Courdavault V. Giglioli-Guivarc’H N. St-Pierre B. Simkin A. J. (2011). Subcellular evidence for the involvement of peroxisomes in plant isoprenoid biosynthesis. *Plant Signal. Behav.* 6 2044–2046. 10.4161/psb.6.12.18173 22080790 PMC3337203

[B10] Cordier H. Karst F. Bergès T. (1999). Heterologous expression in *Saccharomyces cerevisiae* of an *Arabidopsis thaliana* cDNA encoding mevalonate diphosphate decarboxylase. *Plant Mol. Biol.* 39 953–967. 10.1023/A:1006181720100 10344201

[B11] Dassanayake R. S. Cao L. Samaranayake L. P. Berges T. (2002). Characterization, heterologous expression and functional analysis of mevalonate diphosphate decarboxylase gene (MVD) of *Candida albicans*. *Mol. Genet. Genomics* 267 281–290. 10.1007/s00438-002-0648-7 12073030

[B12] Dhingra S. Cramer R. A. (2017). Regulation of sterol biosynthesis in the human fungal pathogen aspergillus fumigatus: opportunities for therapeutic development. *Front. Microbiol.* 8:92. 10.3389/fmicb.2017.00092 28203225 PMC5285346

[B13] Escano C. S. Juvvadi P. R. Jin F. J. Takahashi T. Koyama Y. Yamashita S. (2009). Disruption of the aopex11-1 gene involved in peroxisome proliferation leads to impaired woronin body formation in *Aspergillus oryzae*. *Eukaryot Cell* 8 296–305. 10.1128/EC.00197-08 19136573 PMC2653236

[B14] Espenshade P. J. Hughes A. L. (2007). Regulation of sterol synthesis in eukaryotes. *Annu. Rev. Genet.* 41 401–427. 10.1146/annurev.genet.41.110306.130315 17666007

[B15] Fowler D. M. Cooper S. J. Stephany J. J. Hendon N. Nelson S. Fields S. (2011). Suppression of statin effectiveness by copper and zinc in yeast and human cells. *Mol. Biosyst.* 7 533–544. 10.1039/c0mb00166j 21085730 PMC3138400

[B16] Görög S. (2011). Advances in the analysis of steroid hormone drugs in pharmaceuticals and environmental samples (2004–2010). *J. Pharm. Biomed.* 55 728–743. 10.1016/j.jpba.2010.11.011 21131155

[B17] Grant S. G. Jessee J. Bloom F. R. Hanahan D. (1990). Differential plasmid rescue from transgenic mouse DNAs into *Escherichia coli* methylation-restriction mutants. *Proc. Natl. Acad. Sci. U.S.A.* 87 4645–4649. 10.1073/pnas.87.12.4645 2162051 PMC54173

[B18] Hogenboom S. Tuyp J. M. Koster J. Wanders R. J. Waterham H. R. (2004). Human mevalonate pyrophosphate decarboxylase is localized in the cytosol. *Mol. Genet. Metab.* 81 216–224. 10.1016/j.ymgme.2003.12.001 14972328

[B19] Hu Z. He B. Ma L. Sun Y. Niu Y. Zeng B. (2017). Recent advances in ergosterol biosynthesis and regulation mechanisms in *Saccharomyces cerevisiae*. *Indian J. Microbiol.* 57 270–277. 10.1007/s12088-017-0657-1 28904410 PMC5574775

[B20] Hu Z. Li G. Sun Y. Niu Y. Ma L. He B. (2019). Gene transcription profiling of *Aspergillus oryzae* 3.042 treated with ergosterol biosynthesis inhibitors. *Braz. J. Microbiol.* 50 43–52. 10.1007/s42770-018-0026-1 30637636 PMC6863321

[B21] Jonathan L. David M. (2011). Origins and early evolution of the mevalonate pathway of isoprenoid biosynthesis in the three domains of life. *Mol. Biol. Evol.* 28 87–99. 10.1093/molbev/msq177 20651049

[B22] Kodedova M. Sychrova H. (2015). Changes in the sterol composition of the plasma membrane affect membrane potential, salt tolerance and the activity of multidrug resistance pumps in *Saccharomyces cerevisiae*. *PLoS One* 10:e0139306. 10.1371/journal.pone.0139306 26418026 PMC4587746

[B23] Kovacs W. J. Olivier L. M. Krisans S. K. (2002). Central role of peroxisomes in isoprenoid biosynthesis. *Prog. Lipid Res.* 41 369–391. 10.1016/S0163-7827(02)00002-4 12121718

[B24] Krepkiy D. Miziorko H. M. (2004). Identification of active site residues in mevalonate diphosphate decarboxylase: implications for a family of phospho transferases. *Protein Sci.* 13 1875–1881. 10.1110/ps.04725204 15169949 PMC2279928

[B25] Lazo G. R. Stein P. A. Ludwig R. A. (1991). A DNA transformation-competent arabidopsis genomic library in agrobacterium. *Biotechnology* 9 963–967. 10.1038/nbt1091-963 1368724

[B26] Lees N. D. Skaggs B. Kirsch D. R. Bard M. (1995). Cloning of the late genes in the ergosterol biosynthetic pathway of *Saccharomyces cerevisiae* —a review. *Lipids* 30 221–226. 10.1007/BF02537824 7791529

[B27] Ma S. M. Garcia D. E. Reddingjohanson A. M. Friedland G. D. Chan R. Batth T. S. (2011). Optimization of a heterologous mevalonate pathway through the use of variant HMG-CoA reductases. *Metab. Eng.* 13 588–597. 10.1016/j.ymben.2011.07.001 21810477

[B28] Maruyama J. Nakajima H. Kitamoto K. (2002). Observation of EGFP-visualized nuclei and distribution of vacuoles in *Aspergillus oryzae* arpA null mutant. *FEMS Microbiol. Lett.* 206 57–61. 10.1111/j.1574-6968.2002.tb10986.x 11786257

[B29] Michihara A. Morita S. Toda K. Akasaki K. Tsuji H. (2008). Mevalonate pyrophosphate decarboxylase is predominantly located in the cytosol of both B16 and B16F10 cells in mouse melanoma treated with lovastatin. *J. Health Sci.* 54 216–223. 10.1248/jhs.54.216

[B30] Michihara A. Sawamura M. Nara Y. Ikeda K. Yamori Y. (1998). Lower mevalonate pyrophosphate decarboxylase activity is caused by the reduced amount of enzyme in stroke-prone spontaneously hypertensive rat. *J. Biochem.* 124 40–44. 10.1093/oxfordjournals.jbchem.a022094 9644243

[B31] Miziorko H. M. (2011). Enzymes of the mevalonate pathway of isoprenoid biosynthesis. *Arch. Biochem. Biophys.* 505 131–143. 10.1016/j.abb.2010.09.028 20932952 PMC3026612

[B32] Nguyen K. T. Ho Q. N. Do L. Mai L. T. D. Pham D. N. Tran H. T. T. (2017). A new and efficient approach for construction of uridine/uracil auxotrophic mutants in the filamentous fungus *Aspergillus oryzae* using *Agrobacterium tumefaciens*-mediated transformation. *World J. Microbiol. Biotechnol.* 33:107. 10.1007/s11274-017-2275-9 28466303

[B33] Nguyen K. T. Ho Q. N. Pham T. H. Phan T. N. Tran V. T. (2016). The construction and use of versatile binary vectors carrying pyrG auxotrophic marker and fluorescent reporter genes for agrobacterium-mediated transformation of *Aspergillus oryzae*. *World J. Microbiol. Biotechnol.* 32:204. 10.1007/s11274-016-2168-3 27804102

[B34] Ohneda M. Arioka M. Nakajima H. Kitamoto K. (2002). Visualization of vacuoles in *Aspergillus oryzae* by expression of CPY-EGFP. *Fungal Genet. Biol.* 37 29–38. 10.1016/s1087-1845(02)00033-6 12223187

[B35] Polakowski T. Stahl U. Lang C. (1998). Overexpression of a cytosolic hydroxymethylglutaryl-CoA reductase leads to squalene accumulation in yeast. *Appl. Microbiol. Biotechnol.* 49 66–71. 10.1007/s002530051138 9487712

[B36] Shao J. Shi G. Wang T. Wu D. Wang C. (2016). Antiproliferation of berberine in combination with fluconazole from the perspectives of reactive oxygen species, ergosterol and drug efflux in a fluconazole-resistant candida tropicalis isolate. *Front. Microbiol.* 7:1516 10.3389/fmicb.2016.01516 PMC5034683 27721812

[B37] Shi L. Qin L. Xu Y. Ren A. Fang X. Mu D. (2012). Molecular cloning, characterization, and function analysis of a mevalonate pyrophosphate decarboxylase gene from *Ganoderma lucidum*. *Mol. Biol. Rep.* 39 6149–6159. 10.1007/s11033-011-1431-9 22203490

[B38] Shoji J. Y. Arioka M. Kitamoto K. (2006). Vacuolar membrane dynamics in the filamentous fungus *Aspergillus oryzae*. *Eukaryot Cell* 5 411–421. 10.1128/ec.5.2.411-421.2006 16467481 PMC1405889

[B39] Simkin A. J. Guirimand G. Papon N. Courdavault V. Thabet I. Ginis O. (2011). Peroxisomal localisation of the final steps of the mevalonic acid pathway in planta. *Planta* 234 903–914. 10.1007/s00425-011-1444-6 21655959

[B40] Sun Y. Niu Y. He B. Ma L. Li G. Tran V. T. (2019). A dual selection marker transformation system using *Agrobacterium tumefaciens* for the industrial *Aspergillus oryzae* 3.042. *J. Microbiol. Biotechnol.* 29 230–234. 10.4014/jmb.1811.11027 30602269

[B41] Takaya K. Higuchi Y. Kitamoto K. Arioka M. (2009). A cytosolic phospholipase A2-like protein in the filamentous fungus *Aspergillus oryzae* localizes to the intramembrane space of the mitochondria. *FEMS Microbiol. Lett.* 301 201–209. 10.1111/j.1574-6968.2009.01818.x 19889028

[B42] Tamura K. Peterson D. Peterson N. Stecher G. Nei M. Kumar S. (2011). MEGA5: molecular evolutionary genetics analysis using maximum likelihood, evolutionary distance, and maximum parsimony methods. *Mol. Biol. Evol.* 28 2731–2739. 10.1093/molbev/msr121 21546353 PMC3203626

[B43] Tanabe Y. Maruyama J. Yamaoka S. Yahagi D. Matsuo I. Tsutsumi N. (2011). Peroxisomes are involved in biotin biosynthesis in aspergillus and arabidopsis. *J. Biol. Chem.* 286 30455–30461. 10.1074/jbc.M111.247338 21730067 PMC3162405

[B44] Veen M. Stahl U. Lang C. (2003). Combined overexpression of genes of the ergosterol biosynthetic pathway leads to accumulation of sterols in *Saccharomyces cerevisiae*. *FEMS Yeast Res.* 4 87–95. 10.1016/s1567-1356(03)00126-0 14554200

[B45] Voynova N. E. Fu Z. Battaile K. P. Herdendorf T. J. Kim J. J. P. Miziorko H. M. (2008). Human mevalonate diphosphate decarboxylase: characterization, investigation of the mevalonate diphosphate binding site, and crystal structure. *Arch. Biochem. Biophys.* 480 58–67. 10.1016/j.abb.2008.08.024 18823933 PMC2709241

[B46] Yamada R. Yoshie T. Wakai S. Asai-Nakashima N. Okazaki F. Ogino C. (2014). Aspergillus oryzae-based cell factory for direct kojic acid production from cellulose. *Microb. Cell Fact.* 13:71. 10.1186/1475-2859-13-71 24885968 PMC4035902

[B47] Zuzana I. Katarína P. Dana T. Roman H. Ivan H. Smith A. R. (2013). The yeast *Saccharomyces cerevisiae* Pdr16p restricts changes in ergosterol biosynthesis caused by the presence of azole antifungals. *Yeast* 30 229–241. 10.1002/yea.2956 23606207

