# Supplementary material for: Mevalonate Diphosphate Decarboxylase MVD/Erg19 Is Required for Ergosterol Biosynthesis, Growth, Sporulation and Stress Tolerance in Aspergillus oryzae
Source: Front Microbiol. 2019 May 16;10:1074. doi: 10.3389/fmicb.2019.01074 (PMC6532591; doi:10.3389/fmicb.2019.01074)
Supplement: Supplementary file 1 [file Data_Sheet_1.docx]

Table S1 Primers used for qRT–PCR

| **Name** | **Forward** | **Reverse** |
| --- | --- | --- |
| *rH* | GACAACATCCAGGGTATCACTAAGC | GGTCTCCTCGTAGATCATGGCA |
| *Erg19* | CTGCCTTCGCCGAGATTAC | CGCGAGAGACATCGTTCATATAG |
| *Erg8* | TGACTCCATCTGAGCCGTGACG | ACCTCTACTGCTCGTGGACACTC |
| *Erg12* | ACCTCTACTGCTCGTGGACACTC | TCAAGGATCGCCTCCGTCACTAG |
| *HMG-A* | GAGGCGACTGCTGGCAATGG | AAGAAGTTGGAGGTTGGCGTGATC |
| *HMG-B* | TGTGCTCAATGCTTCGGCTGAC | GATTGCGGTCTGGATCGAGTCTTG |
| *HMG-C* | TGCTGCTTGCGTTGCGGAAG | CACCACCAACCACACCAACAGAC |
| *HMG-D* | AGAGGTGAGATGACTCCAGGAAGC | GCAGCACGGAGCGTCTATATCG |
| *HMG-E* | AAGAAGGTGCGGCGATGAATGG | CGGCGTCATATTCGGCTGTCAG |

Table S2 Primers used for vector construction

| **Name** | **Forward** | **Reverse** |
| --- | --- | --- |
| RNAi-forward | CCGCTCGAGATGGCTGCTCCTTCTGACAGTAC | R1:AGGGTATGCTTAGAGGTTGTGCTTACGGACTGGGGAAGCTGGT |
|  |  | R2:GTCAGATACTCTGAAGATAGGCCATTAGGGTATGCTTAGAGGTTG |
|  |  | R3:CGAGCTCAATTTCCAGTGATTAGTCTCTTGTGTCAGATACTCTGAAGATAG |
| RNAi-reverse | CGAGCTCGGACTGGGGAAGCTGGTAAAGG | CGCGGATCCATGGCTGCTCCTTCTGACAGTAC |
| AoErg19-DsRed | CATTTCACGTGCCCGTGCTTAAGATGGCTGCTCCTTCTGACAG | GGAGGCCATGATATCCTTAAGGTTGGAGAGAATGTCACCTGTC |
| DsRed- AoErg19 | AAGCTTATGGCTGCTCCTTCTGACAGTACGGTCTTTCG | TCTAGACTACAGGAACAGGTGGTGGCGGCCCTCGGCGC |
| GFP-PTS1 | TGAGCAGACATCACCCTCGAGATGGTGAGCAAGGGCGAGG | TCAGTAACGTTAAGTGGATCCCTACAGACGGGACTTGTACAGCTC |
| AoErg19-pYES2 | CATTTCACGTGCCCGTGCTTAAGATGGCTGCTCCTTCTGACAG | GGAGGCCATGATATCCTTAAGGTTGGAGAGAATGTCACCTGTC |
| MTS-GFP | ATGGCTTCTTCCTTGAGAATCGGAA | CTTGTACAGCTCGTCCATGCCGTGA |
| CPY-GFP | ATGAGAGTTCTTCCAGCTAC | GAACCACTCGCCACCCAACCAGCG |
| GFP-AoVam3 | ATGTCTTTCGACCGTCTTAGTTC | TTATCCAATAGTAGCCGCCAGCACA |
