# Supplementary material for: Mevalonate Diphosphate Decarboxylase MVD/Erg19 Is Required for Ergosterol Biosynthesis, Growth, Sporulation and Stress Tolerance in Aspergillus oryzae
Source: Front Microbiol. 2019 May 16;10:1074. doi: 10.3389/fmicb.2019.01074 (PMC6532591; doi:10.3389/fmicb.2019.01074)
Supplement: Supplementary file 2 [file Data_Sheet_2.docx]

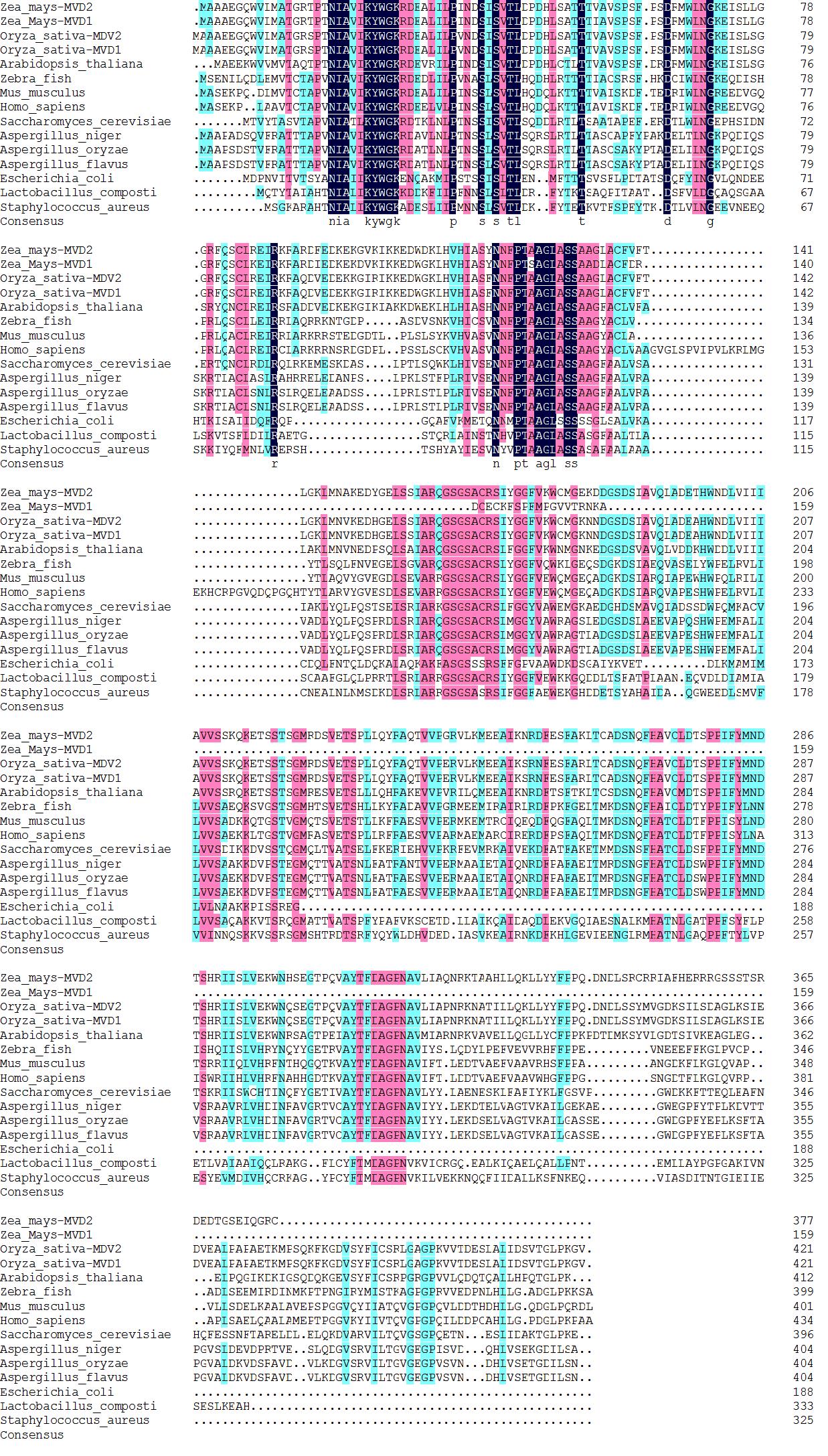


Fig. S1. Comparison of the amino acid sequence of MVD in different species. Comparison of the amino acid sequence of MVD in *Zea Mays*, *Oryza sativa*, *Zebra fish*, *Mus musculus*, *Homo sapiens*, *Arabidopsis thaliana*, *S. cerevisiae*, *A. niger*, *A. oryzae*, *A. flavus*, *Ecoli*, *Lactobacillus composti* and *Staphylococcus aureus*.


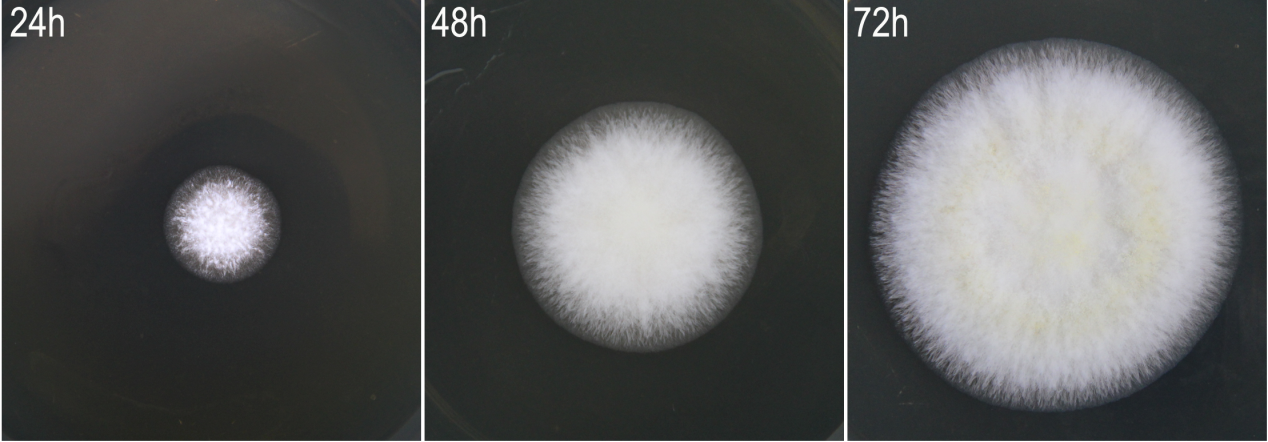


Fig. S2 The morphologies of *A. oryzae* colony after 24, 48 and 72 h cultivation.


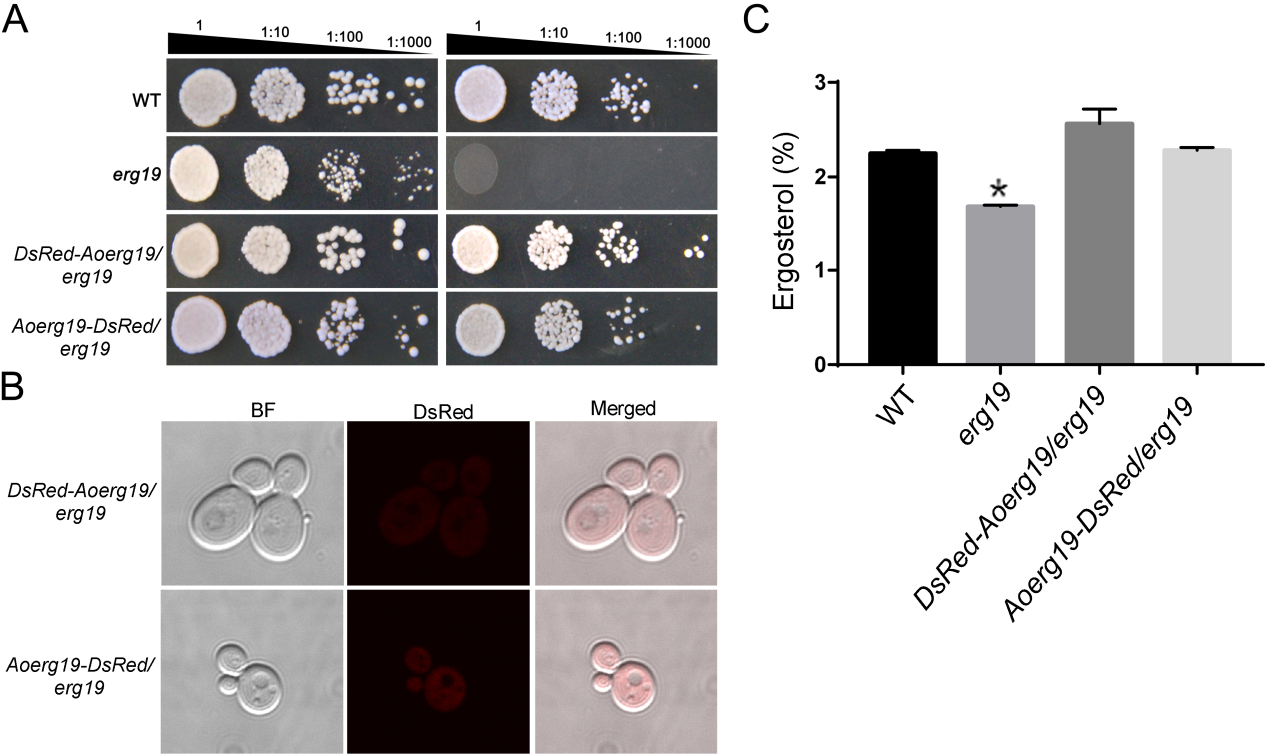


Fig.S3 The phenotypes and ergosterol contents of heterologous expression AoErg19-DsRed and DsRed-AoErg19 in *S. cerevisiae*. (A) Growth of *S. cerevisiae* wild type, *erg19* mutant and *AoErg19-Ds-Red/erg19* and *DsRed-AoErg19/erg19* transformants on YPD and YPG medium. (B) The DsRed fluorescence in *AoErg19-Ds-Red/erg19* and *DsRed-AoErg19/erg19* transformants. (C) The ergosterol contents in *S. cerevisiae* wild type, *erg19* mutant and *AoErg19/erg19* transformant. Asterisks denote significant differences compared with control (P < 0.05).


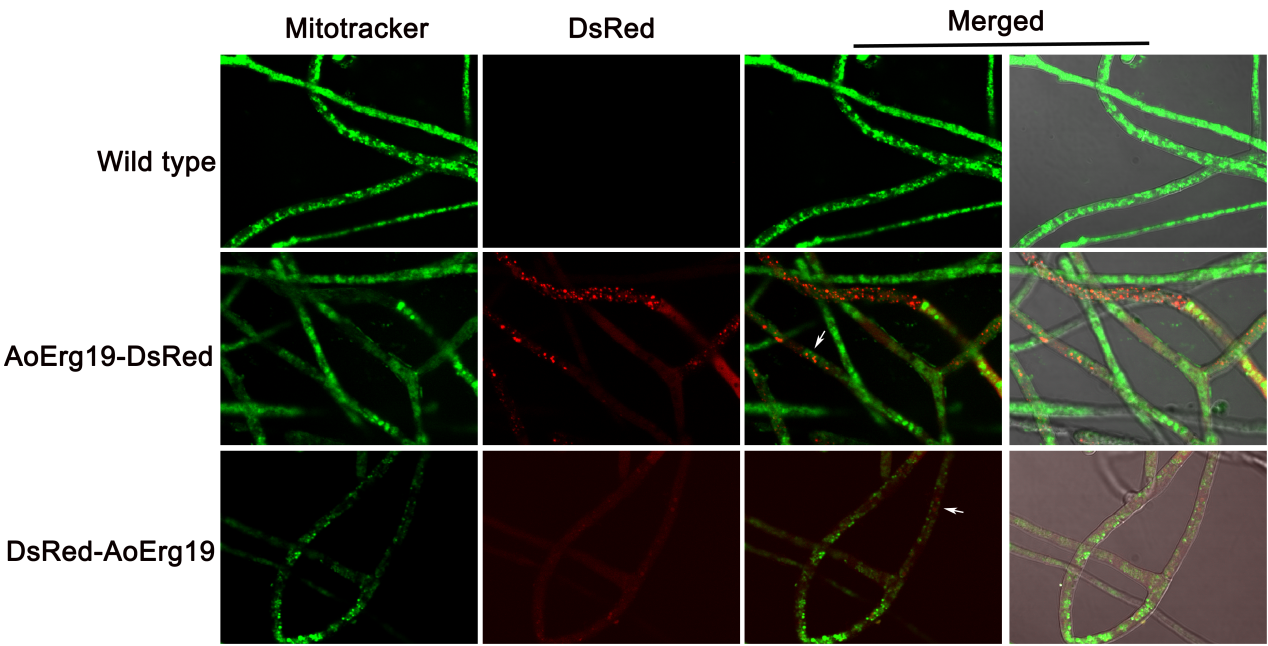


Fig. S4 Co-localization of AoErg19-DsRed/DsRed-AoErg19 with mitochondria by Mito-tracker green staining. From top to bottom are mitotracker stained wild type, AoErg-DsRed and DsRed-AoErg. From left to right are fluorescent image of mitotracker, DsRed, merged image of mitotracker and DsRed, and merged image of GFP, DsRed and bright field (data not show).


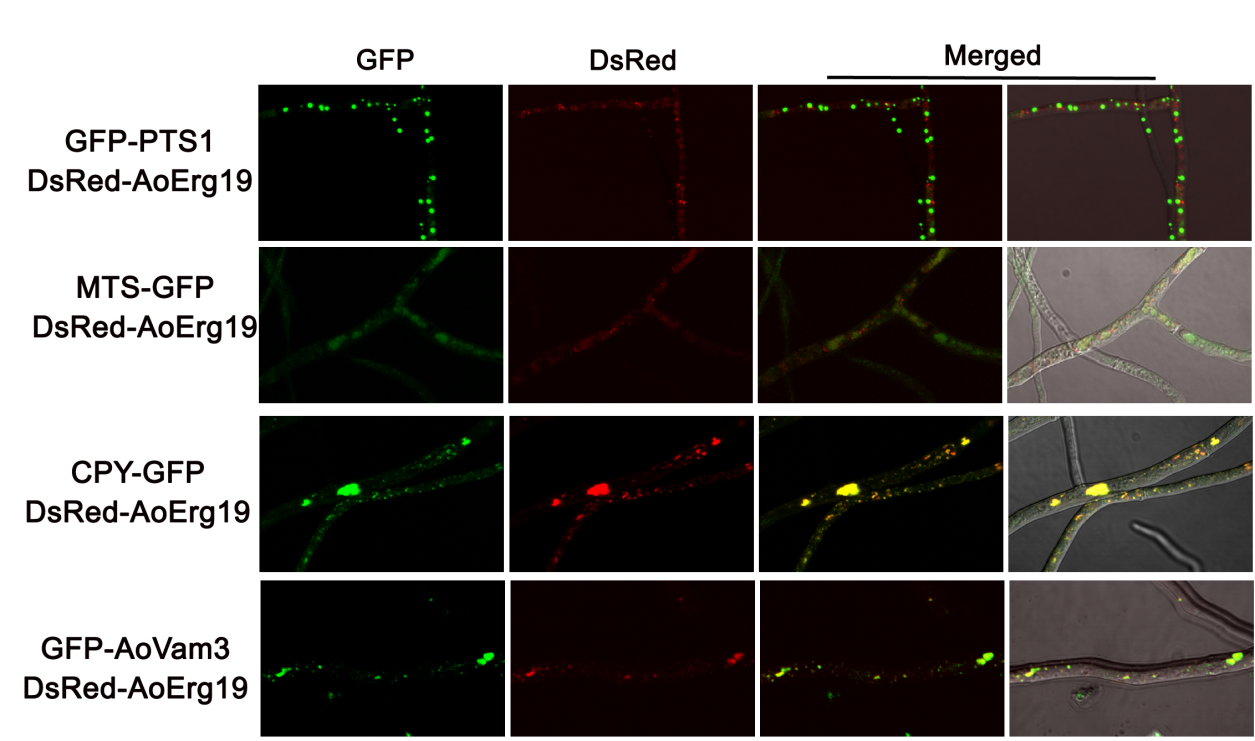


Fig. S5 Co-localization of DsRed-AoErg19 with peroxisome, mitochondria or vacuole. From top to bottom are the co-localization of DsRed-AoErg19 with GFP-PTS1, MTS-GFP, CPY-GFP and GFP-AoVam3. From left to right are fluorescent image of GFP, DsRed, merged image of GFP and DsRed, and merged image of GFP, DsRed and bright field (data not show).


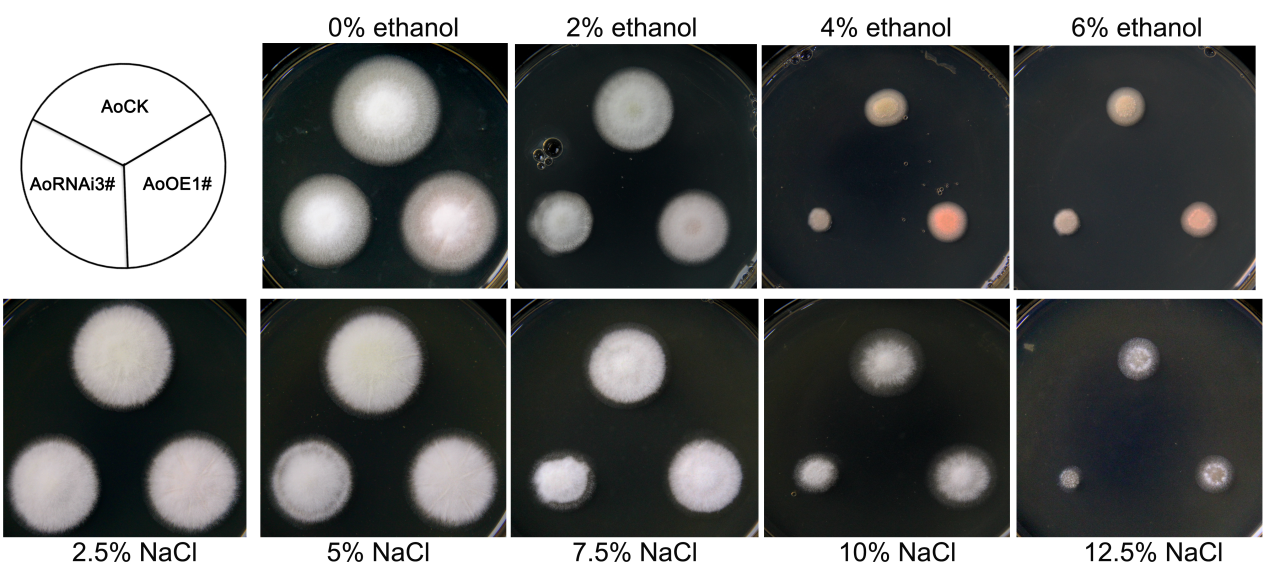


Fig. S6 Colony morphologies under ethanol and NaCl stress conditions. Colony morphologies of control, AoOE1# and AoRNAi3# stains under ethanol (up panel) and NaCl (up panel) stress conditions.
